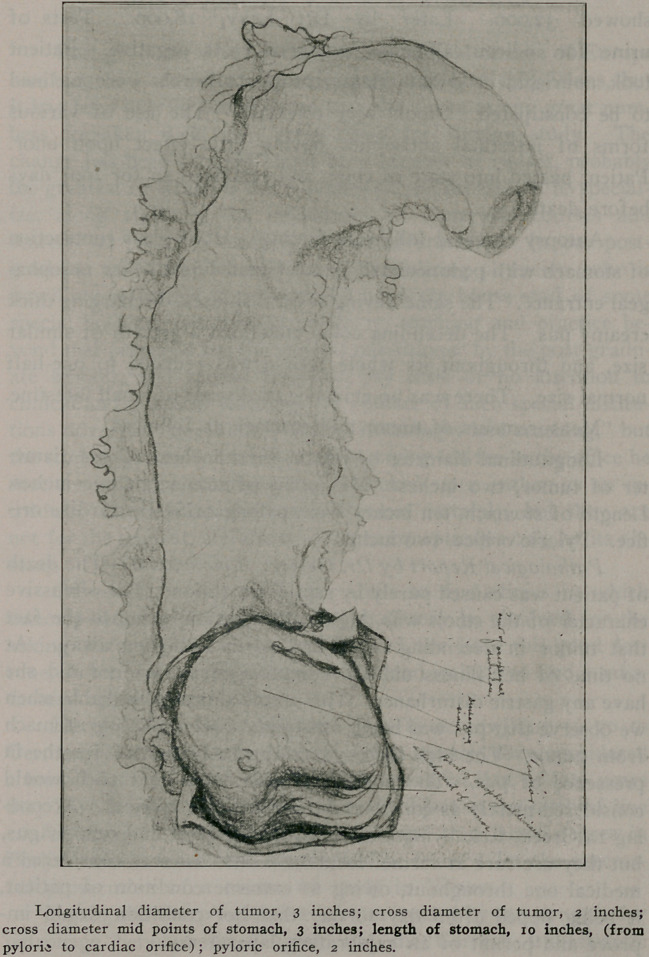# Report of a Case of Suppurative Leiomyoma of Stomach

**Published:** 1908-09

**Authors:** Whatley W. Battey

**Affiliations:** Assistant Professor Anatomy and Clinical Surgery, Medical Department University of Georgia, Augusta, Ga.


### REPORT OF A CASE OF SUPPURATIVE LEIOMYOMA OF STOMACH. HOUR GLASS CONTRACTION OF SAME.

BY WHATLEY W. BATTEY, JR., M. D., ASSISTANT PROFESSOR ANATOMY AND CLINICAL SURGERY, MEDICAL DEPARTMENT UNIVERSITY OF GEORGIA, AUGUSTA, GA.

Mrs. B., age 62, occupation housewife. On January 26, 1907, patient complained of lassitude, loss of appetite and constipation, temperature 102 1-5 degrees Fahrenheit. She was seen by Dr. Battey, Sr., who administered a cathartic and put patient to bed on a liquid diet. Condition was regarded as one of autotoxemia. After bowels moved freely abdominal distention and tympany was materially relieved. Temperature touched normal. Patient was up and around third day following visit. One week after first visit patient had a relapse, was put to bed and treated for original conditions. Stools at this time were very offensive, temperature ranged 100 in a. m., between 102 and 103 1-5 in p. m. At times a. m., temperature was normal. For several days afternoon temperature touched 99 degrees. Abdomen was very much distended, pulse ranged from 100 to 120. Absence of nausea or vomiting, no tenderness or abdominal rigidity.

Condition was thought to be typhoid. Diazo reaction negative. Widal negative. Haemoglobin estimate 75 per cent.

I was called in consultation on February 22, 1908. The general make up of patient to me was typhoid, though she had never had, nor did not present rose-spots. On morning of February 23, on palpating abdomen, I discovered a mass in epigastrium which was movable, but owing to marked distention the exact location of growth was somewhat confusing. Owing to cachetic appearance of patient and presence of mass, I advanced the diagnosis of cancer of intestine, stomach omentum or transverse colon. At another examination of abdomen I did not find mass first felt, and I was inclined to believe it to have been a foecal accumulation in transverse colon. At a third examination of abdomen I felt the mass distinctly and was thoroughly satisfied with diagnosis.

Longitudinal diameter of tumor, 3 inches; cross diameter of tumor, 2 inches; cross diameter mid points of stomach, 3 inches; **length of stomach, 10 inches, (from** pyloric to cardiac orifice); pyloric orifice, 2 inches.

Several other consultants were called in, who in the presence of marked abdominal distention could not locate mass and agreed that symptoms were typhoid, owing to fact that 1 to 20 Widal done by Dr. Levy was positive. Leucocytosis by Dr. Wood showed 32,000. Later by Dr. Levy, 18,000. Tests of urine for indican, albumen, sugar or casts negative. Patient took nourishment without least trouble. Bowels were inclined to be constipated. Stools very offensive. The use of various forms of intestinal antiseptics having little effect upon odor. Patient passed into state of como and remained so for four days before death.

Autopsy revealed following finding: Hour glass contraction of stomach with pedunculated tumor situated just below oesophageal entrance. The same having several sinuses, discharging thick creamy pus. The decending colon contained a growth of similar size, and throughout its whole course was reduced to one-half normal size. There was no evidence of disease of small intestine.

Measurements of tumor and stomach as follows:

Longitudinal diameter of tumor, three inches. Cross diameter of tumor, two inches. Mid point of stomach, three inches. Length of stomach, ten inches from pyloric orifice to cardiac orifice. Pyloric orifice, two inches.

*Pathological Report by Dr. Oertel*: *Leiomyoma.*—The death of patient was caused purely by septic absorption. The offenssive character of the stools was due to putrefaction owing to the fact that tumor in decending colon had partly sloughed away. At no time of her illness did she complain of nausea nor did she have any gastric disturbance. This seems almost incrediable when we observe that pus was being constantly discharged into stomach from tumor. The high leucocytosis can be accounted for the in pressence of pus. The positive Widal shows that such would considered merely as corroborative evidence in typhoid. According to Green, Leiomymata occur in the stomach and oesophagus, but they are rare in farmer location. The case was considered a medical one throughout, owing to extreme condition of patient, it being hoped from day to day that her condition would improve and permit of an exploratory laparatomy.

## Figures and Tables

**Figure f1:**